# Posttranslational modification of differentially expressed mitochondrial proteins in the retina during early experimental autoimmune uveitis

**Published:** 2011-07-06

**Authors:** Sindhu Saraswathy, Narsing A. Rao

**Affiliations:** Doheny Eye Institute, Department of Ophthalmology, Keck School of Medicine of the University of Southern California, Los Angeles, CA

## Abstract

**Purpose:**

Posttranslational modification of proteins plays an important role in cellular functions and is a key event in signal transduction pathways leading to oxidative stress and DNA damage. In this study, we used matrix-assisted laser desorption/ionization- time of flight (MALDI-TOF) to investigate the posttranslational modifications of the differentially expressed proteins in the retinal mitochondria during early experimental autoimmune uveitis (EAU).

**Methods:**

EAU was induced in 18 B10RIII mice with 25 µg of inter-photoreceptor retinoid-binding protein (IRBP) emulsified with complete Freund’s adjuvant (CFA); 18 mice treated with CFA without IRBP served as controls. Retinas were removed from the experimental and control groups on day 7 post immunization; mitochondrial fractions were extracted and subjected to 2 dimentional-difference in gel electrophoresis (2D-DIGE); and the protein spots indicating differential expression were subjected to MALDI-TOF for protein identification and indication of any posttranslational modifications.

**Results:**

Of the 13 proteins found to be differentially expressed by 2D-DIGE (including upregulated aconitase, mitochondrial heat shock protein (mtHsp) 70, lamin-1, syntaxin-binding protein, αA crystallin, βB2 crystallin, along with downregulated guanine nucleotide-binding protein and ATP synthase) nine were found to undergo posttranslational modification. Oxidation was a common modification found to occur on aconitase, mtHsp 70, ATP synthase, lamin-1, βB2-crystallin, guanine nucleotide-binding protein, and manganese superoxide dismutase (MnSOD). In addition, aconitase hydratase, mtHsp 70, guanine nucleotide-binding protein, ATP synthase, syntaxin-binding protein, βB2-crystallin, and lamin-1 were also modified by carbamidomethylation. αA-crystallin had a pyro-glu modification.

**Conclusions:**

Several proteins present in the retinal mitochondria are posttranslationally modified during early EAU, indicating the presence of oxidative stress and mitochondrial DNA damage. The most common modifications are oxidation and carbamidomethylation. A better understanding of the proteins susceptible to posttranslational modifications in the mitochondria at the early stage of the disease may serve to advance therapeutic interventions to attenuate disease progression.

## Introduction

The experimental autoimmune uveitis (EAU) animal model is widely used to decipher the immune and molecular mechanisms leading to intraocular inflammation and subsequent photoreceptor degeneration [1-3]. However, recent studies have revealed that photoreceptor degeneration in the form of photoreceptor mitochondrial DNA damage takes place before inflammatory cell infiltration, and the mechanism for such retinal damage appears to be initiated by photoreceptor mitochondrial oxidative stress [4,5]. Using proteomics, we recently demonstrated mitochondrial oxidative stress leading to altered protein expression of retinal mitochondria during early phase EAU, before inflammatory cell infiltration [3]. Increased levels of manganese superoxide dismutase (MnSOD), mitochondrial heat shock protein (mtHsp) 70, αA-crystallin, and βB2-crystallin were detected in the mitochondria using 2 dimentional-difference in gel electrophoresis (2D-DIGE) coupled with mass spectrometry, suggesting the occurrence of changes in mitochondrial proteomics caused by oxidative stress.

However, the oxidative stress-mediated posttranslational changes that occur in EAU are unknown. Posttranslational modification of proteins plays an important role in cellular functions and is a key event in signal transduction pathways leading to oxidative stress and DNA damage [6-9]. Altered expression of mitochondrial proteins and posttranslational modification of these proteins are known to cause mitochondrial dysfunction [6,10-12]. Mitochondrial dysfunction has also been associated with a wide range of pathological diseases, including neurodegenerative diseases, cancer, diabetes, ischemia, and aging [7,11-14]. Identification of the posttranslational modifications of mitochondrial proteins will aid in further understanding the pathologic effector mechanisms of these diseases and can provide information on therapeutic targets for mitochondrial oxidative stress-related diseases. In this study, we used matrix-assisted laser desorption/ionization- time of flight (MALDI-TOF) to investigate the posttranslational modifications of differentially expressed proteins in the retinal mitochondria during the early phase of EAU.

## Methods

### Induction of uveitis and extraction of retinal mitochondria

Animal care and use was in compliance with University of Southern California guidelines and with the Association for Research in Vision and Ophthalmology Statement for the Use of Animals in Ophthalmic and Vision Research. EAU was induced in 8-week-old B10.RIII mice (Jackson Laboratory, Bar Harbor, ME). Twenty-five micrograms of inter-photoreceptor retinoid-binding protein peptide SGIPYIISYLHPGNTILHVD in PBS (1X, 137 mM NaCl, 2.7 mM KCl, 4.3 mM Na_2_HPO_4_, 1.47 mM KH_2_PO_4_, pH of 7.4) was emulsified at a ratio of 1:1 (volume/volume) with complete Freund’s adjuvant supplemented with *Mycobacterium tuberculosis* strain H37RA to 2.5 mg/ml as previously described [3]. A total of 300 μl of emulsion was injected subcutaneously in three sites: base of tail and both thighs (EAU group). The control group consisted of B10.RIII mice injected with normal saline. The control group consisted of B10.RIII mice injected with complete Freund’s adjuvant only. Retinas were isolated from two groups of 36 B10RIII mice. Each group consisted of 18 day 7 EAU and 18 control mice. In our study the mice were sacrificed by intracardiac injection of sodium pentabarbital, the eyes were enucleated, and the retina was immediately dissected and flash frozen in liquid nitrogen. Extraction of mitochondrial protein was then performed immediately. The mitochondrial proteins were separated from the cytosolic proteins from the pooled retinas of each group, using the mitochondria/cytosol fractionation kit (BioVision Inc., Mountain View, CA) [3,15]. The details of the procedure have been described previously [3]. Suspensions were observed under a microscope to check the efficiency of homogenization. A shiny ring around the cell indicated that the cell was intact. The lysate was spun first for 10 min at 700× g to remove cellular debris and then at 10,000× g for 30 min to pellet the mitochondria. The resulting supernatant was saved as the cytosol portion while the pellet, containing whole mitochondria, was lysed with a mitochondria-specific buffer supplied with the kit. The purity of the fractions was checked by western blot with the mitochondrial marker prohibitin and the cytosolic markers caspase 3 and calpain. The mitochondrial proteins from EAU and control retinas were then subjected to 2D-DIGE followed by mass spectrometric analysis.

### 2D-DIGE and mass spectrometry analysis for the detection of posttranslational modifications of mitochondrial proteins

2D-DIGE was performed by Applied Biomics (Hayward, CA). Briefly, 30 μg each of the protein from the control and EAU mitochondrial fractions were labeled with cyanine dyes (Cy3 and Cy5; Amersham Biosciences, Piscataway, NJ) and the same amount of pooled standard containing equal amounts of control and EAU samples was labeled with Cy2 and used to normalize the Cy3 and Cy5 samples. The details of the 2D-DIGE analysis are described in our earlier report [3]. The three labeled samples were combined and diluted with sample buffer (8 M urea, 4% CHAPS, 20 mg/ml DTT, 2% pharmalytes and a trace amount of bromophenol blue). The Immobilized pH gradient strips (linear range:13 cm, pH 3–10) were rehydrated overnight with destreak solution and rehydration buffer (7 M urea, 2 M thiourea, 4% CHAPS, 20 mg/ml Dithiothreitol, 1% Pharmalytes and a trace amount of bromophenol blue). The manufacturer’s (Amersham Biosciences, Pittsburg, PA) protocol was followed for isoelectric focusing. The IPG strips were then equilibrated in freshly made equilibration buffer 1 (50 mM Tris-HCl, pH 8.8, containing 6 M urea, 30% glycerol, 2% SDS, a trace amount of bromophenol blue, and 10 mg/ml DTT) with slow shaking, then rinsed in freshly made equilibration buffer 2 (50 mM Tris-HCl, pH 8.8, containing 6 M urea, 30% glycerol, 2% SDS, a trace amount of bromophenol blue, and 45 mg/ml DTT. The IPG strips were then rinsed in the SDS-gel running buffer before they were transferred into gradient SDS-Gel (9%–12% SDS-gel prepared using low fluorescence glass plates) and sealed with 0.5% (w/v) agarose solution (in SDS-gel running buffer). Image scans were performed immediately following the SDS–PAGE using Typhoon TRIO (Amersham Biosciences) according to manufacturer’s recommendations. Cy2-, Cy3-, and Cy5- labeled images of each gel were acquired using excitation/emission values of 488/520, 523/580, and 633/670 nm, respectively. The scanned images were then analyzed by Image Quant software (version 5.0, Amersham Bioscience). Protein spot abundance and statistics for the two samples were performed automatically using extended data analysis software (DeCyder software, version 6.0, Amersham Biosciences, Piscataway, NJ). Proteins from the EAU samples that showed an increase or decrease of 20% compared to the control were selected for further analysis. The differentially expressed proteins were then identified using MALDI-ToF/MS analysis [3] and were later studied for posttranslational modifications. MALDI-ToF/MS analysis was performed by Applied Biosystems (Foster City, CA).

## Results

### 2D-DIGE and mass spectrometry for the identification of modified proteins in retinal mitochondria during early experimental autoimmune uveitis

**Figure 1:**
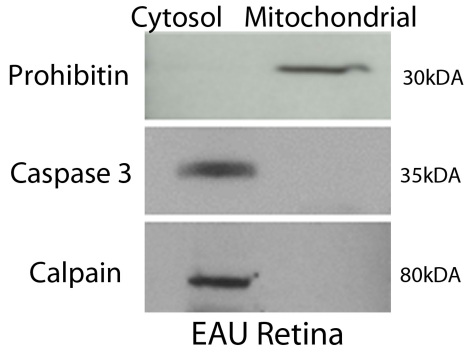
Purity of mitochondrial fractions from early experimental autoimmune uveitis retina. Mitochondria and cytosol were fractionated from the retinas of control (nonimmunized) and experimental autoimmune uveitis (EAU) mice. The purity of each fraction was tested using a mitochondrial marker, prohibitin, and two cytoplasmic markers, caspase 3 and calpain, by western blot analysis. There were no interorganelle contaminants.

**Figure 2:**
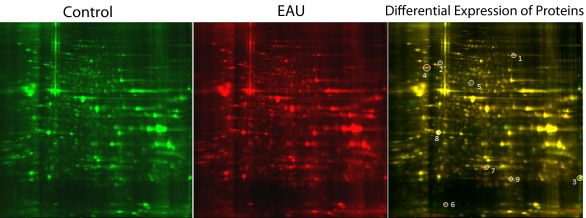
Differential expression of mitochondrial proteins in early experimental autoimmune uveitis retina. On day 7 after immunization, mitochondria were isolated from retinas of B10.RIII mice induced with experimental autoimmune uveitis (EAU) and from control animals. The EAU and control samples were then differentially labeled with cyanine dyes (Cy3 and Cy5) and resolved using a single 2D gel. An internal standard containing equal amounts of each mitochondrial sample labeled with Cy2 was also used. The two images from both samples were overlaid, and the gel was scanned using a highly sensitive typhoon imager and processed by analysis software. The numbered spots were the differentially regulated proteins in EAU samples and these spots were picked for Mass Spectrometry analysis. The differentially expressed proteins were identified by MALDI-TOF/MS.

Before 2D-DIGE analysis, the mitochondrial and cytosolic fractions were checked by western blot analysis for purity by their specific markers prohibitin, caspase 3, and calpain [3]. The results showed that interorganelle contaminants were negligible (Figure 1). Statistical analysis performed from two individual DIGE experiments on 1,000 protein spots revealed 13 proteins that were differentially expressed by 20% fold change in the mitochondria of early EAU compared with matching controls [3]. A similar differential expression of proteins was observed in the two sets of experiments. These differentially expressed proteins were identified by mass spectrometry (Table 1). The ion scores of each identified peptide and the number of peptides matching in each spot are shown in Table 2. Posttranslational modifications of these altered proteins were also determined by MALDI-ToF MS (Table 3). Of the 13 proteins found to be differentially expressed by 2D-DIGE, nine were found to undergo posttranslational modification. Figure 2 shows the spots of differentially expressed proteins which had posttranslational modifications. Most of the modified proteins were oxidized. Aconitase hydratase, mtHsp 70, ATP synthase, MnSOD, βB2-crystallin, and lamin-1 were all oxidized in the early EAU retina. In addition, aconitase hydratase, mtHsp 70, guanine nucleotide-binding protein, ATP synthase, syntaxin-binding protein, βB2-crystallin, and lamin-1 were also modified by carbamidomethylation. αA-crystallin had a pyro-glu modification.

**Table 1:** Diferentially expressed mitochondrial proteins in the retinal mitochondria during early experimental autoimmune uveitis (EAU) identified by MALDI-TOF MS.

**Mitochondrial protein**	**Percent change in early EAU**
Aconitase	24%
mtHsp70	21%
ATP synthase	−100%
Lamin-1	56%
Syntaxin binding protein	33%
αA-crystallin	115%
βB2-crystallin	95%
Guanine nucleotide binding protein	−30%
MnSOD	126%

**Table 2:** Ion scores obtained from MALDI-ToF of each differentially expressed protein in the early experimental autoimmune uveitis (EAU) retinal mitochondria.

**Protein**	**Ion score**	**Ions core C.I.%**	**# of peptides matching**
Aconitase hydratase	97.40	100	8
mtHSP70	80.44	100	8
lamin	91.74	100	7
Syntaxin binding protein	88.79	100	7
ATP Synthase	91.38	100	7
αA-crystallin	83.07	100	5
βB2-crystallin	91.46	100	7
Guanine nucleotide binding protein	107.22	100	13
MnSOD	93.24	100	4

**Table 3:** Post translational modifications of the differentially expressed proteins in the mitochondria of early experimental autoimmune uveitis (EAU) retina.

**Protein**	**Accession #**	**Sequence**	**Modification**
Aconitase	IP100116074	VAMSHFEPSEYIRDVGGIVLANACGPCIGQWDR	M [3]; C [11,14]
mtHsp70	IP100133903	SDIGEVILVGGMTRRPCFSALTVDETYVPK	M [12]; C [3]
ATP synthase	IP100118986	FSPLTANLMNLLAENGRGEVPCTVTTASPLDDAVLSELK	M [9]; C [5]
Lamin-1	IP100230394	LAQALHEMRCQSLTEDLEFR	M [8]; C [1]
Syntaxin binding protein	IP100415403	AAHVFFTDSCPDALFNELVK	C [10]
αA-crystallin	IP100109729	QDDHGYISR	Pyro-Glu
βB2-crystallin	IP100222211	IRDMQWHQRAGSVLVQAGPWVGYEQANCK	M [4]; C [19]
Guanine nucleotide binding protein	IP100120716	IYAMHWGTDSRELAGHTGYLSCCR	M [4]; C [11,12]
MnSOD	IP100109109	HSLPDLPYDYGALEPHINAQIMQLH	M [22]

In the Table, under "Modification", M indicates Oxidation and C indicates Carbamidomethylation.

## Discussion

In the present study, analysis of the altered proteins in the mitochondria of the early EAU retina revealed posttranslational modification. Of the 13 proteins found to be differentially expressed by 2D-DIGE, nine (including upregulated aconitase hydratase, mtHsp 70, βB2-crystallin, αA-crystallin, MnSOD, syntaxin-binding protein, and lamin-1, along with downregulated guanine nucleotide-binding protein and ATP synthase) were found to undergo posttranslational modification. Oxidation was a common modification, which was found to occur on aconitase hydratase, mtHsp 70, MnSOD, βB2-crystallin, ATP synthase, guanine nucleotide-binding protein, and lamin-1. However, mtHsp 70, ATP synthase, syntaxin binding protein and lamin-1 were also modified by carbamidomethylation in the EAU retina. αA-crystallin had a pyro-glu modification in the EAU mitochondria. These results clearly indicate the presence of oxidative stress and mitochondrial DNA damage followed by mitochondrial dysfunction. The results confirm our earlier finding of photoreceptor mitochondrial oxidative stress and DNA damage in early EAU [3-5,16].

Furthermore, mitochondrial dysfunction contributing to oxidative damage in early EAU may also trigger apoptosis since this organelle is intimately involved in initiating programmed cell death [17-21]. However, there was no apoptosis at this stage as previously reported and this might be due to the presence of crystallins in the mitochondria [1,3,15].

This study is a continuation of our previous report [3] in which we had shown differentially modified proteins in the mitochondria of EAU mice retina compared to normal controls. PCR analysis also demonstrated a decrease in the mRNA expression of ATP synthase and increase in mtHsp 70. Western blot analysis of these proteins further confirmed the PCR results. The levels of MnSOD, ATP synthase, aconitase, αA crystallin, and β-crystallin by western blot analysis was also confirmed by 2D-DIGE and mass spectrometry. MnSOD and aconitase were upregulated in EAU retinal mitochondria, whereas ATP synthase was significantly downregulated compared to normal retinal mitochondria. In this study we extended the proteomic details to investigate whether the differentially modified proteins were posttranslationally modified during early EAU. Since this study was conducted to detect differential expression of protein profile in the EAU retinal mitochondria, the levels and posttranslational modifications of proteins, if present in adjuvant injected or normal animal retina, could not be determined.

Mitochondria have fundamental roles in many cellular processes, including energy metabolism via the oxidative phosphorylation system, the Kreb's cycle, and β-oxidation of free fatty acids [22,23]. Critical steps for heme biosynthesis, ketone body formation and urea degradation takes place in the mitochondria [24-26]. Recent evidence implicates mitochondrial involvement in cellular signaling pathways through modulation of intracellular calcium stores, production of reactive species, and the interaction of nitric oxide on mitochondrial functions, such as respiration and biogenesis [7,27]. Given these important roles of the mitochondrion, it is not surprising that alterations in mitochondrial function are thought to play key roles in the development of human disease.

Posttranslational modifications are tissue and disease specific and modify the function and localization of mitochondria proteins and enzymes. Thus, any interpretation of mitochondrial events in disease should include a careful examination of translational and posttranslational modifications of the mitochondrial proteome. As a source for the formation and targeting of modifications mediated by reactive oxygen and nitrogen species (ROS/RNS), the mitochondrion is recognized as a critical site in cellular responses to oxidative and nitrosative stress [27]. While numerous mechanisms of oxidant-induced injury have been identified, the impact of oxidants on the overall content of mitochondrial proteins, the mitochondrial proteome, have not been studied in detail in EAU. In addition to detecting differential expression of proteins in the mitochondria, it is critical to detect the posttranslational modification of proteins as they play a vital role in cells undergoing oxidative damage [10]. Posttranslational modification of proteins determines their tertiary and quaternary structures and regulates their activities and functions by causing changes in protein activity, their cellular locations, and their dynamic interactions with other proteins, thus contributing to the neurodegenerative diseases [9].

Several studies have also demonstrated the involvement of free radicals in various neurodegenerative diseases where the reactive oxygen species can promote multiple forms of oxidative damage, including protein oxidation [9,10,28]. Protein oxidation rapidly contributes to oxidative stress by directly affecting cell signaling, cell structure, and various enzymatic processes of the cell [29]. In the present study, we used proteomics to identify protein targets of oxidation in the mitochondria of EAU retina. Key mitochondrial proteins, such as ATP synthase and aconitase hydratase, and the key antioxidant MnSOD were oxidized during early EAU.

Exposure of mitochondrial proteins to oxidative stress leads to the posttranslational modification of amino acid residues, which can potentially alter protein structure and/or induce a permanent loss in function. In our present study, modification of cysteine residue was detected in the amino acid sequences of aconitase hydratase, mtHsp 70, ATP synthase, lamin-1, βB2-crystallin, and guanine nucleotide-binding protein. Similarly, recent work has suggested that oxidants can induce reversible modifications, specifically at cysteine residues, which may function either to modulate protein function in response to stress or to protect cysteines from irreversible modifications or “overoxidation” [30]. Thus, oxidative, nitrosative, or alkylation reactions can trigger signaling cascades that result in activation of genes involved in cellular stress responses [30,31]. Whether posttranslational modification to mitochondrial proteins plays a role in the adaptive response of the cell to stress is not known. Because alterations in the redox status of protein thiols are typically critical in regulating a protein's function, the identification of these proteins and the type of modifications present is of significant interest [32].

Posttranslational modifications are generally associated with loss of function and may lead to either the unfolding or degradation of the damaged proteins or to aggregation, leading to accumulation as cytoplasmic inclusions as observed in age-related neurodegenerative disorders [33,34].

Oxidative modification of key mitochondrial proteins, like ATP synthase and MnSOD, may lead to their inactivation and to mitochondrial dysfunction with decreased energy supply, ultimately contributing to cellular damage [28,35]. Despite the well established role of ROS/RNS in EAU, the protein targets that are oxidatively modified by elevated ROS remain elusive. To date, no prior study has characterized the oxidatively modified proteins in the retinal mitochondria during early EAU. Early EAU was chosen intentionally for this study to identify the early signs of oxidative modifications that may lead to mitochondrial dysfunction before a secondary wave of damage is inflicted by infiltrating leucocytes during the late phase of EAU. Numerous mitochondrial proteins involved in energy supply and electron transport system, molecular chaperone activity, and anti-oxidant defense have also been demonstrated to be oxidized and s-nitrosylated in the liver during ischemia repurfusion (I/R) injury and in ethanol-exposed animals, often leading to decreased activity/function [14].

Several studies have also demonstrated the involvement of free radicals in various neurodegenerative diseases where the reactive oxygen species can promote multiple forms of oxidative damage, including protein oxidation. Oxidative modification of critical enzymes, like ATP synthase, leads to inactivation of these enzymes, more ROS leakage with reduced efficiency of oxidative phosphorylation, and decreased ATP synthesis [11,35]. In a study similar to ours, Suh et al. used mass spectrometry to identify oxidized mitochondrial proteins like Hsp 60 and aldehyde dehydrogenase in alcohol-exposed human hepatoma cells and in mouse liver by [8]. Many key mitochondrial enzymes involved in energy supply, fat metabolism, cellular defense, and chaperones were also identified as being oxidatively modified proteins in the liver during ischemic reperfusion and thus may lead to dysfunction and injury of the liver [8,36].

Our present study also revealed carbamidomethylation modification in mtHsp 70, laminin, syntaxin-binding protein, and ATP synthase. This finding suggests inactivation of these proteins and dysfunction leading to increased oxidative stress and mtDNA damage. Similarly the pyro-glu modification in the αA-crystallin protein suggests its altered function. The role of this modification has not been studied in detail; such a modification might modulate αA-crystallin to protect the photoreceptors from apoptosis.

Further studies to identify the role of each posttranslational modification detected in the mitochondrial proteins during the early phase of uveitis and in the development of disease will help us to delineate the mechanism of disease onset and progression in uveitis.
